# Inflammatory milieu and role of epigenetic modifications in high-grade serous ovarian cancer

**DOI:** 10.1186/s13148-026-02080-6

**Published:** 2026-02-12

**Authors:** Satarupa Pradhan, Shama Prasada Kabekkodu, Sangavi Eswaran, Naveena A. N. Kumar, Dinesh Upadhya, Sanjiban Chakrabarty, Vasudha Devi

**Affiliations:** 1 Department of Pharmacology, Kasturba Medical College, Manipal Academy of Higher Education, Manipal, 576104 India; 2 Department of Cell and Molecular Biology, Manipal School of Life Sciences, Manipal Academy of Higher Education, Manipal, 576104 India; 3 Department of Surgical Oncology, Kasturba Medical College, Manipal Academy of Higher Education, Manipal, 576104 India; 4 Centre for Molecular Neurosciences, Kasturba Medical College, Manipal Academy of Higher Education, Manipal, 576104 India; 5 Department of Public Health Genomics, Manipal School of Life Sciences, Manipal, 576104 India; 6 Division of Pharmacology, Department of Basic Medical Sciences, Manipal Academy of Higher Education, Manipal, 576104 India

**Keywords:** HGSOC, Chronic inflammation, Inflammatory mediators, Epigenetics, Chemoresistance

## Abstract

High-grade serous ovarian cancer (HGSOC) is often diagnosed at advanced stages (III/IV), with approximately 80% of patients experiencing relapse due to therapeutic resistance. The disease progression is largely influenced by a dynamic tumor microenvironment (TME), which is marked by sustained inflammation, immune evasion, and epigenetic reprogramming. This review investigates the dual role of inflammatory pathways and epigenetic alterations in driving HGSOC progression and chemo-resistance. A comprehensive literature search of articles from 2000 to 2025 was conducted across PubMed, Google Scholar, and Research Rabbit using search terms including “HGSOC,” “epigenetics,” “inflammation,” and “chemoresistance.” Of 1,166 identified publications, 593 peer-reviewed studies comprising original research, clinical trials, meta-analyses, and reviews were critically analyzed. Findings reveal that chronic inflammation in the TME enhances tumor proliferation, immune suppression, epithelial-mesenchymal transition, and metastasis through cytokines, interferons, and chemokines. Epigenetic mechanisms such as DNA methylation, histone modifications, miRNA and lncRNA contribute to tumor plasticity and treatment failure. Emerging therapies, including histone deacetylase inhibitors, DNA methyltransferase inhibitors, and immune checkpoint inhibitors, anti-inflammatory drugs demonstrate potential in overcoming resistance when used in combination. Integrative treatment strategies that target both inflammatory signaling and epigenetic dysregulation offer a promising avenue for improving patient outcomes. Further clinical exploration of such combination therapies is warranted to address the urgent need for effective interventions in HGSOC.

## Background

Ovarian cancer (OC) is the eighth most prevalent cancer diagnosed in women worldwide, with an estimated 20,890 new cases and 12,730 related deaths in 2025 [1]. Approximately one out of 87 women in their lifetime develop cancer, whereas the invasive OC death risk is approximately one in 130. Over the period from 2015 to 2021, the relative survival rate after five years for OC patients was 51.6% [2]. OC can arise from surface epithelial cells, germ cells, or sex-cord stromal cells. Epithelial ovarian cancer (EOC) accounts for 90% of all OC cases. EOCs are divided into two distinct groups according to their growth patterns- Type I: low-grade EOC; Type II: high-grade EOC [3].

High-grade serous ovarian carcinoma (HGSOC) represents the predominant and most aggressive subtype of EOC, comprising approximately 70–85% of cases and classified as a type II tumor [4, 5]. Its clinical lethality arises from rapid transcoelomic dissemination, extensive genomic instability characterized by near-universal *TP53* mutations (> 96%) and homologous recombination (~ 50%) deficiencies, and the absence of effective early detection modalities, which collectively result in advanced-stage diagnosis in most patients [6–8]. Comprehensive genomic profiling identifies four molecular subtypes of HGSOC-mesenchymal, proliferative, immunoreactive, and differentiated each with distinct biological and clinical features [9]. Histopathologically, HGSOC is defined by marked nuclear pleomorphism, desmoplastic stromal response, and extensive subclonal diversification that underpin therapeutic resistance and disease recurrence. Although platinum-taxane chemotherapy achieves high initial response rates (70–80%), relapses occur in up to 80% of patients within two years, primarily due to adaptive chemoresistance driven by drug efflux mechanisms, DNA repair reactivation, and tumor microenvironment (TME) remodeling. Despite the incorporation of targeted therapies such as PARP inhibitors (PARPi) and anti-angiogenic agents into standard treatment regimens, overall survival remains poor, with a five-year survival rate of approximately 40–50%, underscoring the urgent need for early detection strategies and molecularly guided interventions capable of overcoming therapeutic resistance [10].

In HGSOC, repeated exposure to ovulation- and inflammation-associated cues establishes a chronically inflamed, immunosuppressive peritoneal niche that remodels immune surveillance and sustains a cytokine- and chemokine-enriched environment conducive to malignant progression [11]. Persistent inflammation drives epigenetic reprogramming, as cytokine mediated recruitment of DNMT1 and EZH2 promotes DNA hypermethylation and H3K27me3 silencing of tumor suppressors and interferon genes, while NF-κB signaling induces H3K27ac at pro-metastatic enhancers together sustaining immunosuppression, chemoresistance and disease advancement [12, 13]. Within the inflammatory tumor microenvironment, cytokine and prostaglandin signaling orchestrate epithelial-mesenchymal transition (EMT), enhance peritoneal dissemination, and establish a permissive niche for metastasis [14]. Dissecting this reciprocal crosstalk between inflammatory and epigenetic networks is pivotal for identifying early-stage biomarkers and developing precision therapies capable of intercepting HGSOC progression at its molecular roots [5, 15]. Hence, a comprehensive understanding of the inflammatory milieu in HGSOC is essential to identify and modulate therapeutic targets tailored to individual patient profiles, optimizing treatment outcomes.

**Fig. 1:**
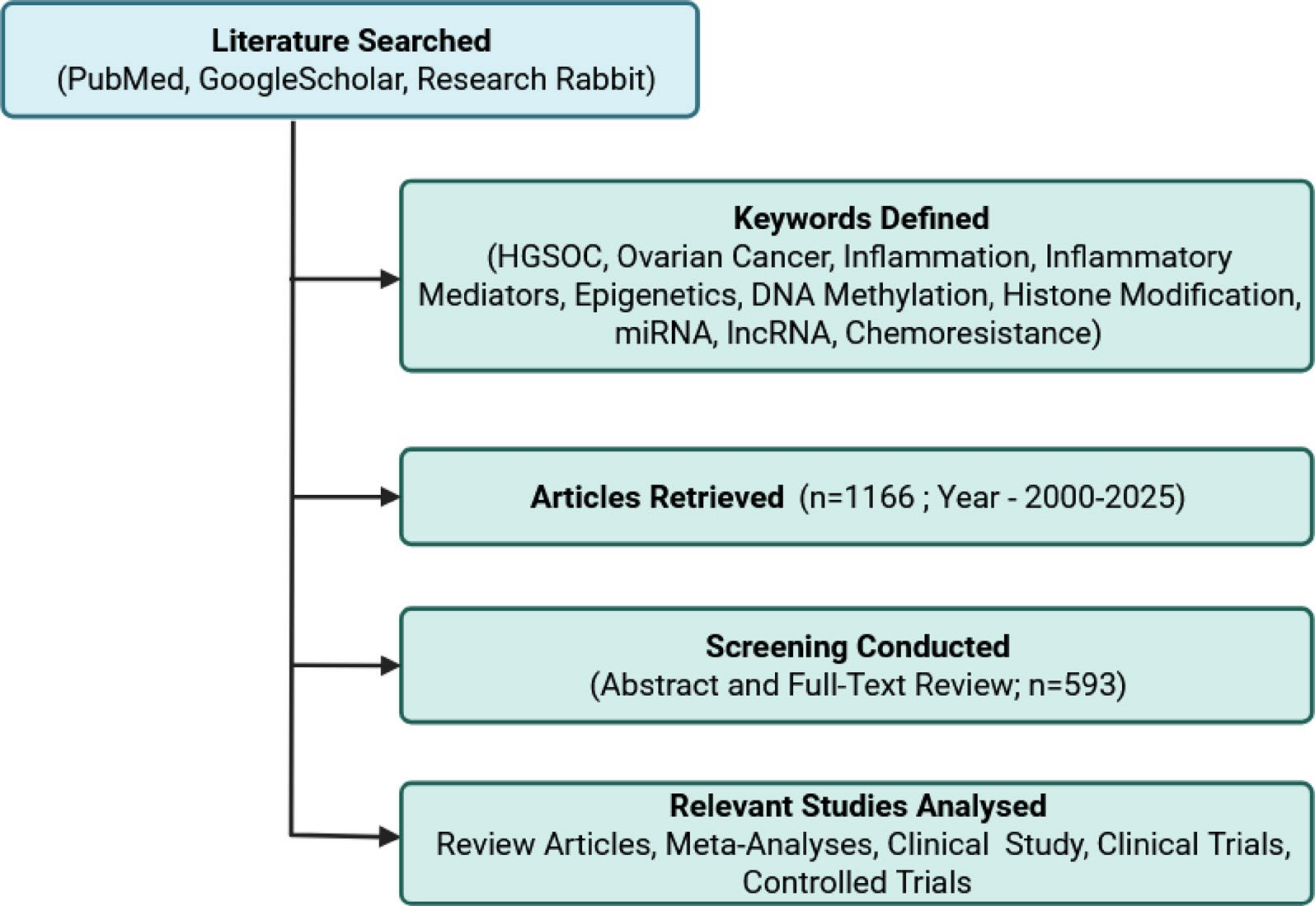
Strategy for the literature search: Database selection, keyword usage and screening of papers. Created in BioRender. https://BioRender.com/39lafch

To investigate the specific interactions among inflammation, epigenetic modifications, and chemoresistance in HGSOC, a curated literature search was carried out. The search included broad terms such as “OC/ovarian cancer,” “HGSOC/high-grade serous ovarian cancer,” “inflammation/inflammatory mediators,” “DNA methylation,” “histone modification,” “miRNA,” “lncRNA,” “epigenetics,” and “therapy resistance/chemoresistance,” pertaining to the inflammation and molecular mechanisms of tumor progression and therapeutic resistance in HGSOC. The review of the literature included a search of PubMed, Google Scholar, Google, and Research Rabbit. Using keyword-based searches, an initial pool of 1,166 articles was found, highlighting the broad spectrum of research activity in this field. Following a rigorous screening process, 593 published research articles in all categories were selected for inclusion in the final analysis. The only publication types included were clinical studies, clinical trials, meta-analyses, and review articles, and all other types of publications were excluded via advanced search filters. A thorough and multifaceted understanding of the research landscape was ensured by this focused approach. This systematic review provides a detailed synthesis of current evidence on the role of inflammatory mediators and epigenetic modifications, including miRNAs, lncRNAs, histone acetylation, and DNA methylation, in the regulation of HGSOC chemoresistance (Fig. 1). By integrating findings from molecular biology, clinical oncology, and epigenetics, this review contributes to a more profound understanding of the underlying mechanisms driving therapeutic resistance and highlights potential targets for innovative therapeutic interventions.

## Inflammation and HGSOC

**Fig. 2:**
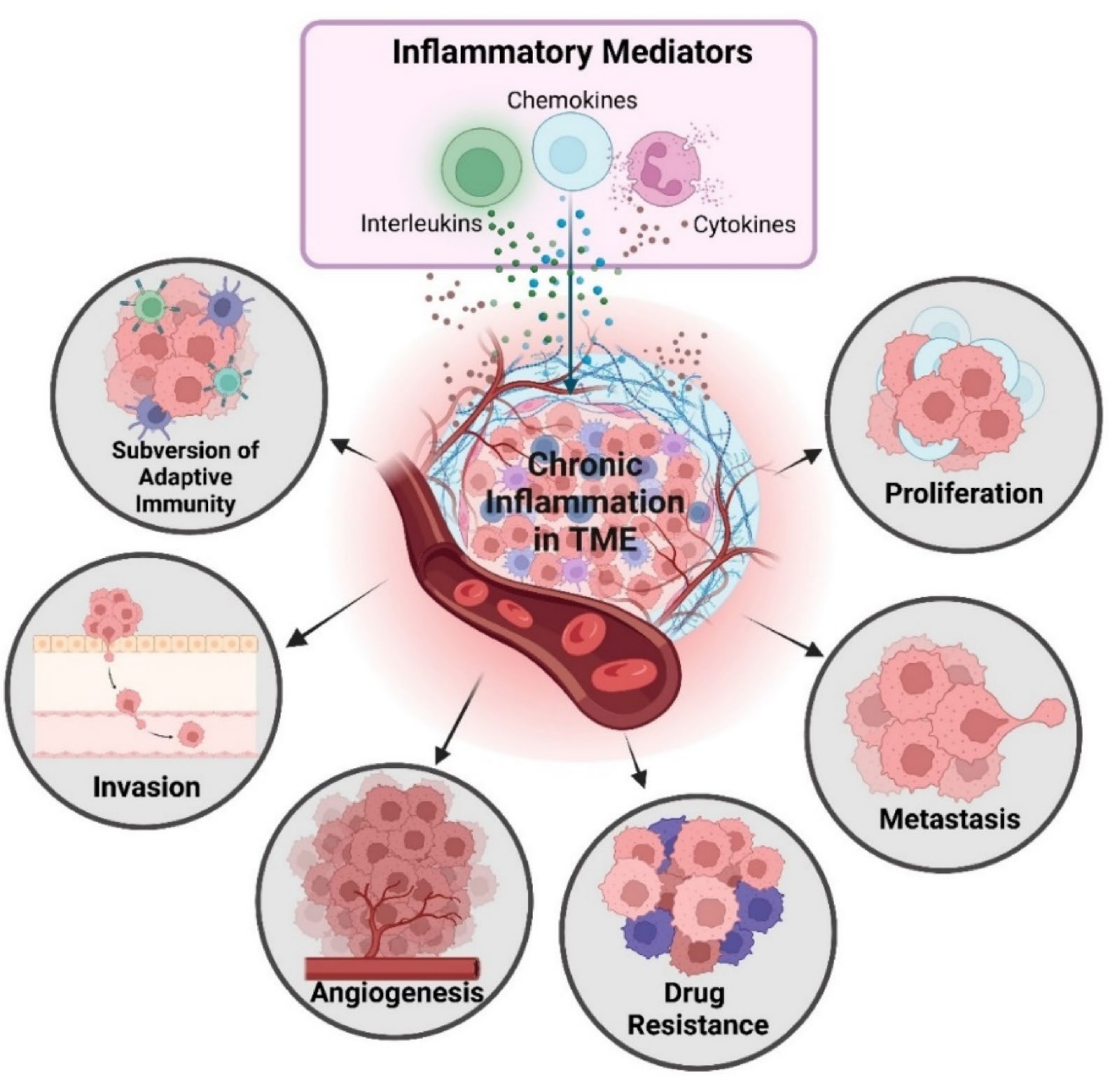
Impact of inflammatory mediators on the tumor microenvironment: Key drivers of HGSOC progression. Chronic inflammation within the TME fosters cancer progression by suppressing adaptive immunity and promoting proliferation, metastasis, drug resistance, and angiogenesis. Created in BioRender. https://BioRender.com/l98b470

Inflammation, a fundamental physiological response to tissue injury characterized by vascular alterations, cytokine signaling, and leukocyte infiltration, is a well-established hallmark of cancer progression [16]. While acute inflammation serves protective and reparative functions, its chronic persistence underlies a spectrum of pathologies including diabetes, atherosclerosis, neurodegeneration, and, notably, EOC, particularly the HGSOC subtype [17, 18]. Within the TME, chronic inflammation cues orchestrate intricate cross talk among immune and stromal cells, leading to the release of interleukins, cytokines, chemokines, and reactive oxygen species (ROS) [19, 20]. These mediators activate NF-κB and STAT3- signalling cascades, that drive cellular proliferation, angiogenesis, invasion, and metastasis, while promoting drug resistance and immune evasion (Fig. 2) [21, 22].

The intrinsic heterogeneity of HGSOC arises from its dual cellular origins predominantly the fallopian tube epithelium (FTE) and, to a lesser extent, the ovarian surface epithelium (OSE) each imparting distinct inflammatory and epigenetic signatures [10]. Recurrent ovulatory injury exposes these epithelia to cytokine- and ROS-enriched follicular fluid, fostering a chronic pro-inflammatory milieu that induces DNA damage, p53 mutation, and chromatin remodeling to initiate serous tubal or ovarian carcinogenesis [11].

Comprehensive transcriptomic profiling further delineates this complexity, identifying four molecular subtypes: immunoreactive, differentiated, proliferative, and mesenchymal as described by Tothill et al. [23]. The immunoreactive subtype shows high CXCL11, CXCL12, and CXCR3 expression, reflecting an immune active or “hot” phenotype enriched in T-cell infiltration and cytokine signaling; the proliferative and mesenchymal subtype, marked by MCM2 and PCNA, FAP, and ANGPTL family expression, represents more immune excluded or “cold” phenotypes associated with stromal activation, EMT and poor prognosis. The differentiated subtype displays more quiescent profile, retaining epithelial features and moderate immune activity [24].

Current evidence suggests that HGSOC lies on an immune continuum, with most tumors exhibiting an immune-cold phenotype characterized by limited cytotoxic activity and immune suppression, while a smaller immunoreactive (hot) subset shows enhanced interferon signaling, active T-cell infiltration, and favorable clinical outcomes, reflecting the immune diversity across its subtypes [25].

## Key inflammatory mediators in HGSOC

**Fig. 3:**
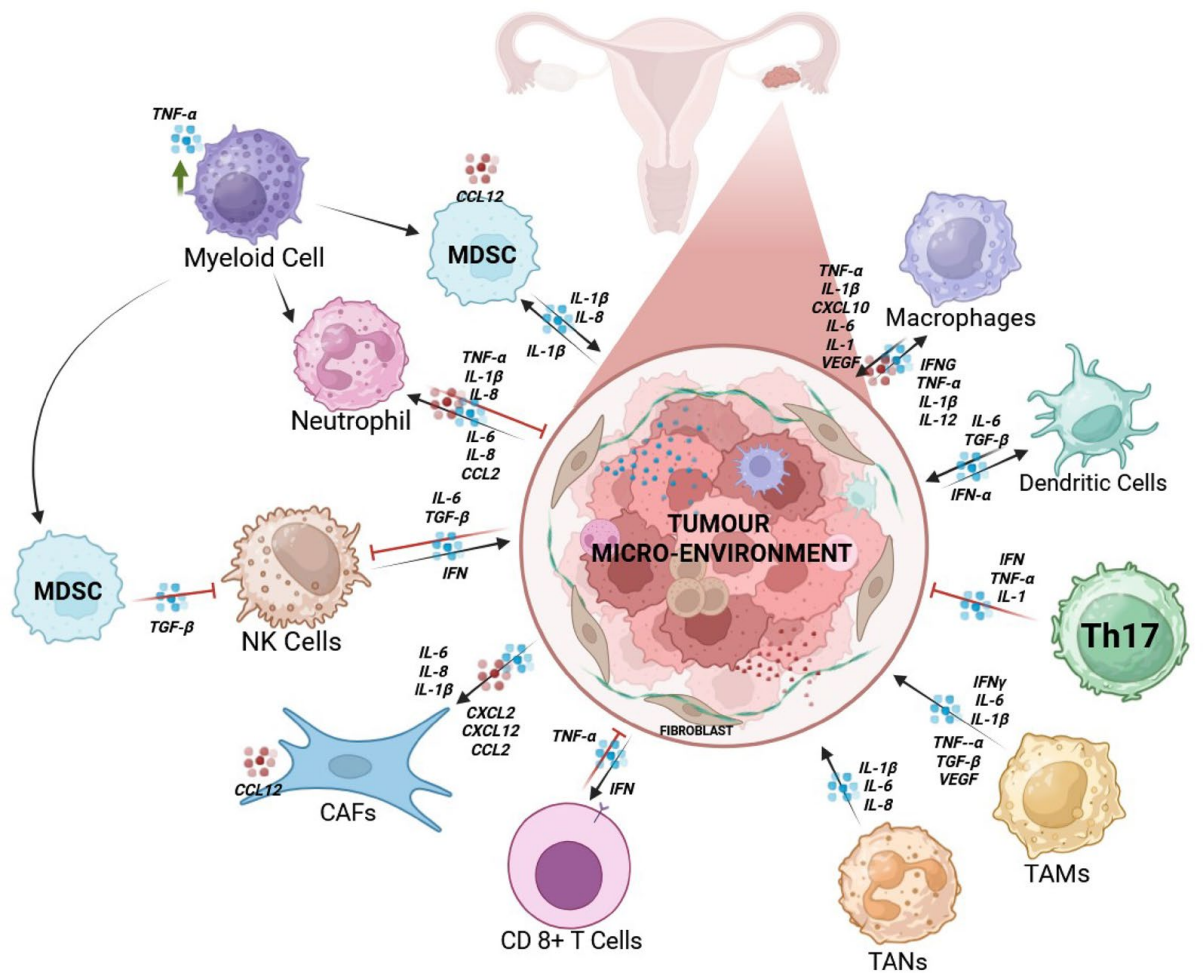
Key inflammatory mediators and their interactions within cells involved in chronic inflammation in the HGSOC tumor microenvironment. This schematic representation illustrates the intricate network of interactions between stromal cells and immune cells within the TME of HGSOC. Various immune cells, including MDSCs, neutrophils, macrophages, dendritic cells, Th1 cells, TANs, TAMs, and CD8⁺ T cells, interact dynamically through inflammatory mediators. Key cytokines, chemokines, and interleukins (e.g., CXCL2, CXCL10, CXCL12, CCL2, TNF, IL1, IL6, IL8, IFNG, VEGF and TGFB) facilitate immune modulation, tumor proliferation, angiogenesis, and immunosuppression. NK cells and CAFs also contribute to this inflammatory environment, which accelerates tumor growth and resistance to treatment. Created in BioRender*.* https://BioRender.com/6hgy89e

Key inflammatory mediators including cytokines, chemokines, and interleukins play pivotal roles in orchestrating the inflammatory milieu that underpins HGSOC pathogenesis [21, 26]. Cytokines act as pleiotropic signaling molecules that modulate immune and stromal cell activation, chemokines govern leukocyte recruitment and spatial organization within the TME, and interleukins regulate both pro- and anti-inflammatory cascades that influence tumor-immune dynamics [27]. Persistent dysregulation of these mediators establishes a pathological feedback loop where tumor and stromal cells engage in reciprocal paracrine signaling, perpetuating cytokine and chemokine production in the TME. This central inflammatory hub, drives EMT, angiogenesis, and immune evasion, while dynamically remodeling the TME to enhance tumor proliferation, peritoneal dissemination, and therapeutic resistance, underscoring its pathways as high-yield targets for disrupting pro-tumorigenic signaling and improving treatment outcomes (Fig. 3) [28].

### Cytokines

#### Transforming growth factor beta (TGFB)

TGFB maintains an angiogenic phenotype and initiates proinflammatory TME. It is produced by neutrophils and macrophages and is essential for the development and spread of cancer [29]. It has three isoforms that use Smads to initiate signaling: TGFB1, TGFB2 and TGFB3 [30]. In the early stages of tumors, TGFB slows tumor growth; however, in later stages, it stimulates angiogenesis, invasion, and proliferation [31–33].

In HGSOC, TGFB plays a significant role in key processes, such as tumor progression, metastasis, angiogenesis, and chemoresistance. TGFB-induced proteins such as TGFB support immune evasion and tumor growth by creating an immunosuppressive TME [34]. TGFB1 dysregulated extracellular matrix (ECM) genes are linked to immune modulation, EMT, and stromal contributions to mesenchymal signatures in HGSOC [35, 36]. EMT is driven by TGFB signaling and is linked to poor prognosis and drug resistance [37]. Additionally, TGFB influences tumor-infiltrating immune cells, such as macrophages, which promote tumor invasion and angiogenesis and interact with other inflammatory molecules, including cytokines and immune cells [38]. TGFB mediated metabolic reprogramming and its crosstalk with WNT signaling impact immune responses and tumor metabolism, highlighting potential therapeutic limitations [39]. Recent studies have shown that TGFB also plays a pivotal role in linking the hypoxic environment to the progression and chemoresistance of HGSOC through a novel molecular mechanism involving the deubiquitinase USP9X and HIF-2α [40].

#### Interferons (IFNs)

IFNs play essential roles in inflammation, immunity, and cancer. IFNs are categorized into 3 types: Type I (e.g., IFNA and IFNB), Type II (IFNG), and Type III (e.g., IFNL1 and IFNL2). Each of these families binds to specific receptors, such as IFNAR, IFNGR, and IFNLR, to activate the JAK-STAT signaling pathway [41]. In cancer, IFNs play dual roles: they increase antitumor immunity and promote tumor survival under chronic inflammation [42]. Chronic IFN signaling promotes tumor adaptation and therapy resistance by selecting IFN-insensitive clones (e.g., with JAK1/2, STAT1, or IRF1 defects), increasing PD-L1 expression via STAT1-BCL3, and recruiting immunosuppressive Regulatory T cells (Tregs) and myeloid-derived suppressor cells (MDSCs) [43]. IFNs act as antitumor mediators, blocking tumor growth by inducing cell-cycle arrest and apoptosis, and enhancing immune visibility through upregulation of MHC-I/II and antigen-processing genes (TAP1/2) [44]. They also recruit and activate NK cells, CD8⁺ T cells, and Th1 cells, promoting a pro-inflammatory microenvironment that correlates with better outcomes, particularly in IFN-high or *BRCA1*-mutant tumors and can enhance responses to radiotherapy, oncolytic viruses, and checkpoint inhibitors [45].

IFNs enhance the immune response against cancer cells in HGSOC by activating ISG15. A higher survival rate is linked to elevated ISG15 levels, which stimulate CD8⁺ lymphocyte infiltration. ISGylation of carcinogenic proteins by ISG15, including ERK of the MAPK pathway, activates CD8⁺ T lymphocytes and NK cells and inhibits the progression of OC [46]. ISG15 also functions in modifying the TME to enhance immune responses since its expression enhances the production of proinflammatory cytokines and induces the activation of immunological checkpoints such as PD-L1. Interferons are critical mediators of the immune response, and therapeutic strategies leveraging PARPi to potentiate interferon signaling pathways have demonstrated considerable promise in augmenting immunotherapeutic efficacy and improving clinical outcomes in HGSOC [47, 48]. A global gene expression analysis revealed that IRF1 (interferon regulatory factor 1) and its mechanistic pathway are associated with platinum resistance in OC [49].

#### Tumor necrosis factor (TNF)

TNF is a proinflammatory cytokine involved in inflammation, ovulation and restoration of the corpus luteum. TNF plays multiple roles in cancer [50–54]. TNF and its receptors, TNFR1 and TNFR2, are overexpressed in ovarian cancer tissues compared with normal tissues. Significant levels were also detected in the ascites of ovarian cancer patients [55–57]. Autocrine TNF signaling in OC, which is mediated via TNFR1, activates a proinflammatory and proangiogenic gene network including IL1A, IL6, CCL2, CXCL8, CXCL12, MIF, M-CSF, and VEGFA through increased mRNA stability and feedback loops, promoting peritoneal dissemination, neovascularization, and sustained tumor progression [57, 58]. TNF also sustains IL17A expression in CD4⁺ T cells via TNFR1, which drives the recruitment of myeloid cells into TME. This reinforces a chronic inflammatory loop characterized by elevated expression of IL23R, IL1R, TGFB1, and NF-κB signaling components, collectively enhancing immune evasion, neutrophil activation, and metastatic capacity within the peritoneal cavity [59]. TNF binds to its receptors and induces tumor proliferation, angiogenesis, and EMT. TNF synergizes with TGFB to drive endothelial-to-mesenchymal transition (EndMT) via sustained Smad2/3 signaling and the upregulation of TGFBR1, TGFBR2, activin A, and integrin αv. EndMT-derived cells secrete TGFB2 and activin, reinforcing mesenchymal traits and inducing EMT in cancer cells, thereby promoting tumor progression [60]. TNF exhibits a context-dependent duality in ovarian cancer: chronic signaling sustains inflammation, IL6/STAT3-driven TNFR2⁺ Treg expansion, and immune evasion, while acute activation triggers TNFR1-linked NF-κB stress and apoptotic death in tumor cells. In HGSOC, it shows growth inhibition and NF-κB-dependent cytokine induction, contrasting with the proliferative, NF-κB-independent response of clear-cell and endometrioid types. Thus, fine-tuning TNF signaling to favor TNFR1 cytotoxicity while restraining TNFR2/STAT3 inflammation could redefine its role from an inflammatory driver to a precision immunotherapeutic target in HGSOC [61, 62].

### Interleukins

#### Interleukin 6 (IL6)

IL6 is included in the class of protumorigenic cytokines of the cytokine family. IL11, IL27, IL31, leukemia inhibitory factor (LIF), oncostatin M (OSM), and ciliary neurotrophic factor (CNTF) are critical protumor cytokines that bind to the interleukin 6 receptor IL6R, leading to the activation of the STAT3 signaling pathway. This activation then causes the transcriptional upregulation of the cyclin D1 and D2 genes, which encode important proteins that drive the cell cycle and cell division. These proteins are involved in the regulation of tumor growth and metastasis through various cells [63]. HGSOC requires the growth-modulating signaling molecule IL6. One diagnostic evidence for HGSOC is elevated blood IL6 levels, which are associated with chronic stages of the disease [64]. IL6 stimulates the Ras-ERK and PI3K-Akt pathways to increase the proliferation and survival of cells [65]. IL6 plays a role in tumor biology by preventing tumor senescence [66, 67] and acts as a growth factor involved in angiogenesis and EMT [68–70]. IL6 is overexpressed in the brain, liver, bone marrow, and lungs as cancer metastasizes [71–73]. IL6 is also negatively correlated with poor prognosis in patients with OC [74–77]. It promotes ascites development and tumor cell invasion by activating the MAPK-ERK-Akt pathways, which in turn stimulate the NF-κB pathway, stimulating proliferation and facilitating cell cycle progression [78]. In ascites, M2 macrophages release IL6 [79], which increases invasiveness by inducing the expression of metal matrix proteins [80], suppresses the ability of IL2 to limit immunological responses [81], and promotes proliferation through STAT3 [78]. IL6 contributes to a relative increase in angiogenesis inside the TME by collaborating with STAT3 to drive angiogenesis in the ovaries via trans-signaling from soluble IL6RA [82, 83]. By activating STAT3, IL6 creates an environment that is protumorigenic and encourages the survival, growth, and enrichment of tumor cells; hence, it is linked to a poor prognosis and chemoresistance [84, 85] [84, 85]. In the TME, IL6 released by cancer-associated fibroblasts and macrophages aids immune suppression and increases the invasive characteristics of tumor cells [86]. In mouse models that mimic human HGSOC, anti-IL6 therapies can successfully modify the immune landscape and tumor response [87].

#### Interleukin 8 (IL8)

IL8 belongs to the C-X-C chemokine family and is expressed in ovarian cysts and preovulatory follicles [88]. Individuals with OC have higher levels of IL8 than healthy normal controls do [89, 90]. Compared with those from patients with benign gynecological disorders, ascites from OC patients has higher levels of IL8 [91]. Elevated IL8 expression has been linked to a poor prognosis in OC patients [90]. IL8 activates Akt and ERK [92] to upregulate cyclins B1 and D1, promoting the proliferation of EOC cells. Nevertheless, few studies have indicated that IL8 suppresses EOC growth by increasing neutrophil infiltration [93, 94]. Additionally, IL8 is essential for angiogenesis [95]; it enhances the synthesis of VEGF, MMP2, and MMP9 in OC cells and promotes angiogenesis in rat models [92]. In mouse models of EOC, growth and angiogenesis are inhibited by IL8, suggesting that IL8 is a therapeutic target that mediates tumor progression [94, 96].

IL8 is an essential factor in the proliferation and prognosis of HGSOC. Elevated IL8 levels are linked to poor outcomes, increased tumor proliferation, angiogenesis, and immune suppression within the TME. IL8 is involved in neutrophil recruitment and NET formation, which is linked to improved survival in some cases [97]. Cytokine profiling of OC ascites highlights the role of IL8 in disease progression and chemoresistance [84, 98]. In HGSOC, IL8 has been identified as a significant biomarker and potential target for treatment [64, 86, 99].

#### Interleukin 1 (IL1)

IL1 is reported to be upregulated in various cancer types, such as breast, colon, lung, and pancreatic cancer and melanoma, and is correlated with a poor prognosis [100]. Tumor cells secrete IL1, which has a mitogenic impact and promotes the synthesis of TGFB and IL6 [101, 102]. T helper 17 (Th17) cells, tumor-associated macrophages (TAMs), tumor-associated neutrophils (TANs), regulatory B (Breg) cells and MDSCs are examples of immunosuppressive cells in the TME that produce IL1, which aids in immune escape mechanisms and carcinogenesis [103].

Studies on the role of IL1A in HGSOC have shown its significant effects on tumor biology and prognosis. IL1A plays a crucial role in the inflammatory responses of *BRCA1*-deficient ovarian malignancies, producing both tumor-intrinsic immunoreactivity and resistance mechanisms [104]. Innate immune chemokines and cytokine networks, including IL1A, are crucial in HGSOC lipopolysaccharide-based treatment methods [105]. Proteomic studies have also associated IL1A with the recurrence of OC, demonstrating the significant role of cytokines within the TME [106]. In patients with EOC and HGSOC, IL1A is a known biomarker of the response to weekly paclitaxel therapy [107].

### Lipid-derived and eicosanoid mediators

#### Prostaglandins

Prostaglandins are produced by the cyclooxygenases COX1 and COX2, which metabolize arachidonic acid and are essential for regulating uterine blood flow and oocyte maturation [108–110]. COX1 and COX2 are overexpressed in patients with OC, which is linked to increased angiogenesis, cell proliferation, and overall malignancy [110, 111]. COX1 and COX2 are key enzymes that mediate acute inflammation. COX2-derived prostaglandins stimulate MDSCs to produce CXCR4 and SDF1/CXCL12, as well as to migrate into OC ascites, which leads to immunosuppression and facilitates tumor formation [112]. In a mouse model of EOC with p53, Rb, and PTEN deletions, along with a KRASG12D mutation, COX1 levels were elevated, indicating that COX1 could serve as a biomarker alongside being a therapeutic target [113]. Inhibition of COX1 in EOC cells results in decreased synthesis of prostacyclin, which in turn increases apoptosis, thereby slowing tumorigenesis [114].

Prostaglandins in HGSOC are important for tumor development, inflammatory processes, and metastasis. An increased concentration of prostaglandin E2 (PGE2) and COX1 and COX2 enzymes is associated with increased cellular proliferation, angiogenesis, and poor prognostic outcomes [115, 116]. Further investigations have shown the role of these prostaglandins in the TME, including immunosuppression, worsening glucose metabolism, and enhancing invasiveness [117, 118]. The expression of prostaglandin D2 also has predictive significance in the context of HGSOC [119]. Targeting COX enzymes and prostaglandin-related pathways has been shown to improve treatment outcomes and detect HGSOC [120, 121]. Collectively, advances in understanding the role of HGSOC prostaglandins and their pathways suggest that they may serve as important markers and therapeutic targets [122].

#### Phospholipid lysophosphatidic acid (LPA)

LPA functions as an activator of the Edg receptor. LPA aids in the synthesis of IL6 and IL8 by the corpus luteum and is present in ovarian follicular fluid [123, 124]. LPA is a growth factor released by OC cells [125–129]. Patients with OC have higher levels of LPA not only in the ascitic fluid but also in the plasma. This contributes to the progression of the disease by exacerbating the upregulation of MMP2 and COX2 [130, 131]. LPA binds to LPA2 receptors and activates NF-κB and AP1, which in turn induces IL-6 and IL-8 synthesis in the O cell line. It also induces the ROS-dependent phosphorylation of Akt and ERK [132, 133].

LPA is important for cell survival and HGSOC progression. In patients with OC, elevated plasma and ascites levels of LPA increase the production of IL6 and IL8 and VEGF expression via Sp-1 and c-Myc, as well as the assembly of focal adhesions, all of which promote angiogenesis and the spread of cancer [134–138]. Additionally, LPA enhances tumor cell endurance and apoptosis suppression in HGSOC cells [139].

### Chemokines

Chemokines, a subtype of cytokines, are associated with OCs and are known to cause chemotaxis in cells, mostly leukocytes. In combination with its receptor, CXCR4, CXCL12 migrates cancer cells and promotes the development of tumors. While chemokines were first identified as regulating leukocyte trafficking at the site of inflammation, they influence all cell types, including cancerous cells. On the invasive front, cancerous cells can modify chemokine-receptor networks to gain control over leukocyte entry and regulate tumor growth. Chemokines are known to play a role in angiogenesis [140].

Owing to their multifaceted role, chemokines play crucial roles in the formation and survival of HGSOC, facilitating tumor dissemination through cancer cell migration and generating an enabling tumor growth environment. Elevated levels of chemokines, such as CXCL12, and their receptor, CXCR4, all contribute to increased tumor aggressiveness and metastasis. When HGSOC patients are divided into *TP53* mutation status groups, the chemokine network has a significantly distinct impact on overall survival [141]. Chemokines facilitate immune evasion by regulating the recruitment and activation of immune cells, which suppress immunity within the TME [25]. Tertiary lymphoid tissues expressing certain chemokine signatures are associated with improved prognosis in patients with HGSOC [142]. In HGSOC, the chemokine CXCL10 is known to act as an antagonistic chemokine with anticancer activity [143]. The therapeutic targeting of chemokines and their receptors via lipopolysaccharide-based therapeutics decreases the tumor burden while enhancing the immune response in the host [105]. The TME of HGSOC is heavily influenced by chemokines and affects both patient chemosensitivity and patient prognosis [144, 145]. Elevated expression of chemokine receptors, such as CXCR4, has been linked to increased proliferation and migration of tumor cells, which is mediated by ligands such as CXCL12 [146].

### Growth factors

#### Fibroblast growth factor (FGF)

FGF family comprises 18 ligands and four tyrosine kinase receptors (FGFR1-FGFR4), which regulate proliferation, survival, angiogenesis, and tissue repair in healthy tissues [147]. In OC, aberrant activation of FGF-FGFR signalling contributes to tumorigenesis, angiogenesis, and stromal remodelling, promoting aggressive biological behaviour [148]. FGF2 is significantly upregulated in platinum-resistant OC and has been identified as a top chemoresistance-associated hub gene, supporting tumor adaptation to cytotoxic stress [149]. FGFR overexpression further amplifies downstream signalling through MAPK/ERK and PI3K/AKT pathways, increasing survival and resistance to apoptosis in OC cells [150, 151]. Silencing FGFR2 has been shown to markedly decrease cisplatin IC₅₀ values and induce apoptosis, demonstrating its role in mediating chemoresistance in OC cells [152].

In HGSOC, FGF ligands and receptors play prominent roles in chemoresistance, tumor progression, and shaping the TME. FGF19 is frequently amplified in HGSOC and promotes cisplatin resistance by activating the p38 MAPK pathway and inducing pro-survival autophagy, while FGF19 knockdown reduces autophagic markers and restores chemotherapy sensitivity [153]. FGF18 is highly expressed in serous ovarian tumors and enhances angiogenesis, macrophage recruitment, and invasiveness, correlating with aggressive disease and poor prognosis [154]. FGFR4 overexpression supports proliferation and survival in serous ovarian cancer models, and its inhibition results in decreased tumor growth and increased apoptosis, highlighting FGFR4 as a therapeutic vulnerability [155]. Overall, evidence indicates that the FGF-FGFR axis is a major driver of HGSOC progression and chemoresistance, making these pathways promising diagnostic and therapeutic targets.

Several other inflammatory mediators beyond the canonical cytokines and chemokines also play crucial roles in the progression of HGSOC, as outlined in **(**Table 1**)**. VEGF is a prominent mediator that drives angiogenesis and enhances vascular permeability, supporting tumor growth and peritoneal dissemination [181]. Platelet-activating factor (PAF) contributes to HGSOC invasion by promoting EMT and enhancing tumor cell motility within the ascitic microenvironment [182]. Another critical factor, epidermal growth factor (EGF), activates the EGFR signaling cascade, which induces a pro-inflammatory transcriptional program that fuels tumor proliferation and chemoresistance [183]. Other mediators such as IL11 and OSM contribute to STAT3 pathway activation and the maintenance of an immunosuppressive tumor milieu [184, 185]. In summary, in the context of HGSOC, the above associated noncanonical inflammatory mediators act synergistically to promote angiogenesis, immune evasion, and metastasis in HGSOC, underscoring their potential as targets for novel therapeutic interventions [117, 120].

**Table 1:** Key inflammatory and immunoregulatory mediators implicated in HGSOC progression

Mediator	Type / Function	Role in HGSOC	Key References
CCL17	Chemokine	Another M2 TAM-associated chemokine, facilitates trafficking of CCR4-expressing regulatory T cells into some tumors thereby mediating resistance	[156]
CCL18	Chemokine	Promotes EOC metastasis via the mTOR signaling pathway	[157, 158]
CCL2 (MCP-1)	Chemokine	Elevated in serum; predicts progression-free survival; correlated with recurrence risk	[159]
CCL22	Chemokine	In ascites, associated with CCR4 + T regulatory cells, they may help recruit immunosuppressive Tregs	[160]
CX3CL1	Chemokine	Promotes intraperitoneal tumor growth in OC models	[161]
CXCL1	Chemokine	High serum / tissue levels associated with chemoresistance in HGSOC	[159, 162]
CXCL10	Chemokine	In ascites, correlates with CXCR3 + T cells; may regulate immune cell trafficking	[163, 164]
CXCL12	Chemokine	Influences T-cell migration, associated with T cell subsets in ascites, contributes to tumor progression	[165]
CXCL9	Chemokine	Elevated in TME; linked to T-cell infiltration; prognostic relevance	[166, 167]
IFNG	Pro-inflammatory cytokine	Part of systemic inflammatory pattern; may influence anti-tumor immunity but also chronic inflammation	[168, 169]
IL10	Anti-inflammatory cytokine	Induces immunosuppressive macrophages (M2), contributes to protumor microenvironment	[170, 171]
IL12	Pro-inflammatory cytokine	Potent antitumor cytokine; studied in combination immunotherapy (IL12 + ICI) in OC	[172]
IL17A	Pro-inflammatory cytokine	May be involved via Th17-like signaling; recent data suggests interplay of Th17 and mesothelial cells promoting metastasis	[173]
IL4	Cytokine	Associated with immunosuppressive TME; affects macrophage polarization / immune resistance	[174]
IL6	Pro-inflammatory cytokine	Promotes M2 macrophage polarization, contributes to tumor-promoting inflammation, immune suppression	[175, 176]
TGFB	Growth factor / immunosuppressive cytokine	High peritoneal TGFB1 associated with NK cell dysfunction in advanced EOC / HGSOC	[177]
VEGF	Growth factor / pro-angiogenic cytokine	Contributes to inflammation, angiogenesis	[178, 179]
FGF7	Epithelial mitogen	Promotes proliferation of serous ovarian epithelial cells and enhances tumor stromal interactions	[180]

## Role of epigenetics in chronic inflammation in HGSOC

In HGSOC, epigenetic reprogramming acts as a key regulator of transcriptional dynamics, integrating environmental and genetic signals into stable yet reversible modifications of gene expression. Three major epigenetic processes- DNA methylation, histone modification, and non-coding RNA regulation contribute to the molecular heterogeneity, adaptive plasticity, and aggressiveness of HGSOC [5, 186]. These aberrations have been implicated in the silencing of tumor suppressors [187], activation of oncogenes [188], and reprogramming of transcriptional networks that collectively drive tumor progression and therapeutic resistance [189].

Emerging evidence underscores that chronic inflammation and epigenetic alterations function synergistically to form a feedback loop [190]. Inflammatory signaling pathways influence epigenetic regulators, leading to persistent changes in transcriptional activity, while epigenetic alterations, in turn, reinforce inflammatory responses and support a tumor-favorable microenvironment. This interplay shapes a stable yet adaptable system that maintains oncogenic activity and dampens anti-tumor mechanisms [191].

**Fig. 4:**
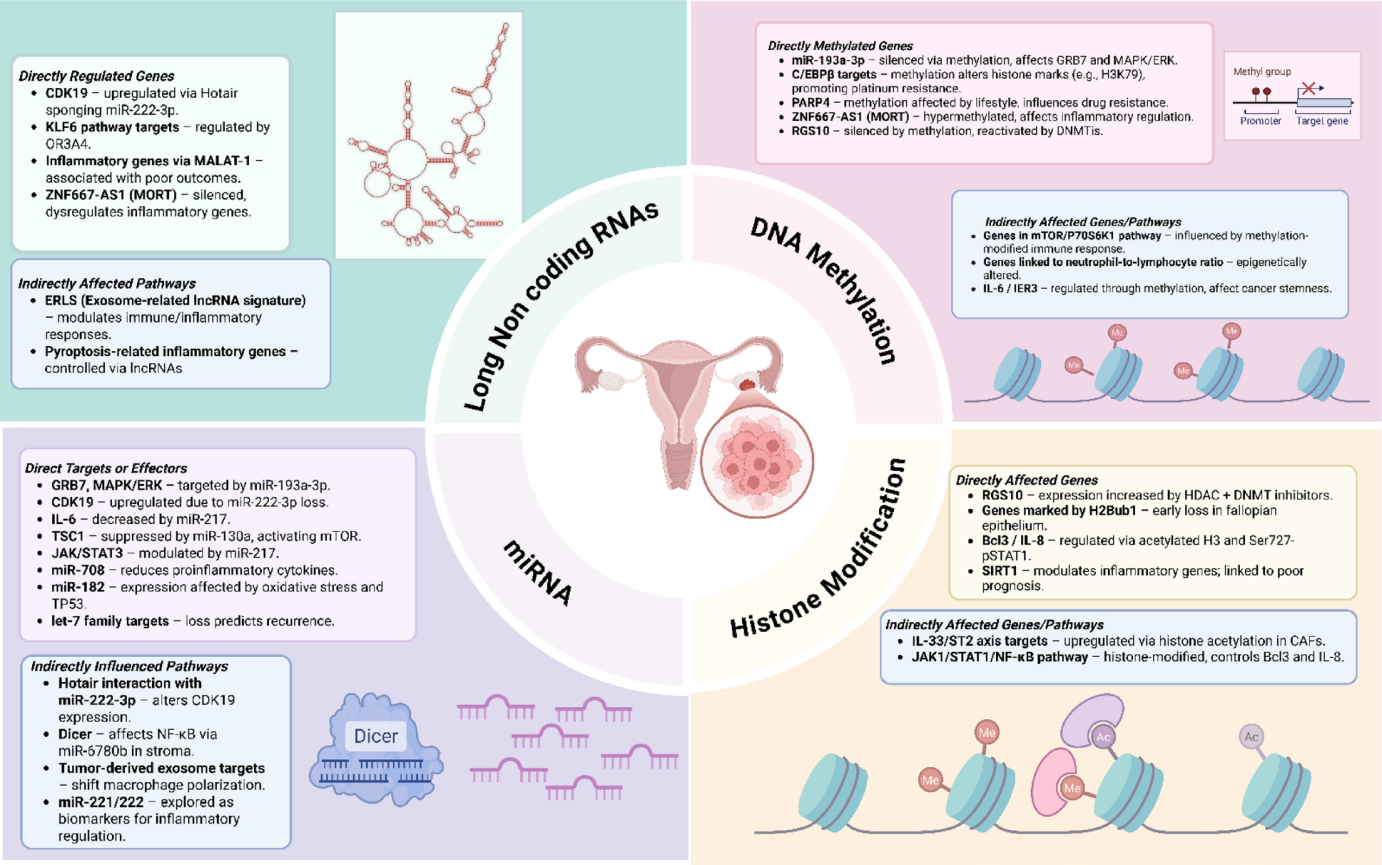
Epigenetic mechanisms regulating chronic inflammation in HGSOC. The schematic diagram summarizing how DNA methylation, histone modifications, noncoding RNA mediated regulation through miRNAs, and lncRNAs influence inflammatory pathways and tumor progression in HGSOC. These key epigenetic regulators have potential to control immune signaling, chemoresistance, and TME dynamics. Created in BioRender. https://BioRender.com/4k9lmjr

The molecular circuitry connecting these processes is reflected in the gene and protein networks influencing inflammatory and epigenetic regulation (Table 2), forming the mechanistic basis illustrated in the integrated model of epigenetic control of inflammation in HGSOC (Fig. 4).

### DNA methylation

DNA methylation is an epigenetic change that modifies gene function and has been linked with the development of many cancers, including HGSOC [217]. The intricate connection between chronic inflammation and DNA methylation in HGSOC is reported in few studies. Chronic inflammation is associated with the progression of neoplasia and affects methylation patterns [218]. A study reported that inflammatory microenvironment may activate certain pathways that increase the expression and function of DNA methyltransferases (DNMTs), thus modifying the methylation of genes relevant to the cell cycle, apoptotic pathways, and immune system functions. This mechanism demonstrates that inflammatory processes may enhance HGSOC malignancy by modifying the epigenetic regulation of critical oncogenes and tumor suppressor genes [219]. Furthermore, DNA methylation of inflammatory genes influences the TME, treatment responses, and contributes to HGSOC chemoresistance [220].

Collective studies shows that, epigenetic alterations, such as hypermethylation of the miR-497 promoter associated with the mTOR/P70S6K1 pathway, which promote cisplatin resistance by modulating the tumor immune response, highlighting the critical role of inflammation in driving chemoresistance [192, 193]. In connection, the reprogramming methylation patterns in inflammatory genes that are regulated by C/EBPβ promotes platinum resistance by altering histone markers such as H3K79, underscoring the importance of inflammation-related epigenetic modifications in mediating chemoresistance [194, 195]. Another study reported that SMAD7 overexpression disrupts the TGFB mediated epigenetic silencing of E-cadherin by promoting promoter demethylation and reducing *TWIST1* binding and H3K9 acetylation, thereby restoring E-cadherin expression, suppressing OC cell invasion. Reactivation of E cadherin silences TGFB targets, and improves disease-free survival in patients [196].Another study explored the role of the methylation-derived neutrophil-lymphocyte ratio as an epigenetic tool for assessing cancer-related inflammation and outcomes and demonstrated its strong predictive value for survival and disease progression [221]. It is evident that inflammation induced DNA methylation alters the expression of genes involved in immune regulation, apoptosis, and drug responsiveness, creating a microenvironment that favors tumor survival and chemoresistance. Therefore, targeting these methylation-driven inflammatory mechanisms is critical for developing effective therapeutic strategies to overcome resistance and enhance patient outcomes.

### Histone modifications

Histone modifications play a critical role in regulating chromatin structure and gene expression. Accumulating evidence shows that histone modifications, particularly acetylation, regulate gene expression in OC. The addition of acetyl groups to histones promotes a relaxed chromatin structure, facilitating the transcription of genes involved in cancer progression [222]. Histone deacetylase (HDAC) inhibitors (HDACis) have shown promise in modulating gene expression and enhancing anti-tumor responses in OC. While preclinical studies highlight their ability to sensitize OC cells to chemotherapy, especially platinum-based agents, by altering gene expression profiles, clinical trials have revealed that toxicity, particularly from pan-HDAC inhibition, limits their utility. Consequently, selective HDAC inhibition is now considered a more favorable approach because of its potential to enhance therapeutic efficacy while minimizing adverse effects [223].

Early histone modifications, such as loss of H2B monoubiquitylation (H2Bub1), driven by RNF20 depletion, occur early in HGSOC and promote oncogenic behavior by increasing chromatin accessibility and activating immune pathways such as IL-6 signaling. These changes enhance fallopian tube epithelial cell migration and clonogenic growth, contributing to tumor initiation and progression [198]. SIRT1, another key epigenetic regulator, engages in regulating inflammatory genes and is linked to poor prognosis and treatment resistance. SIRT1 can bypass *BRCA1* deficiency and reduce the efficacy of PARPi, making it a potential target for modulating inflammation-related pathways and improving treatment outcomes [199, 200]. IFNG induces Bcl3 expression through the JAK1, STAT1 and NF-κB pathways, leading to the release of proinflammatory chemokines such as IL8. The targeting of Ser727-phosphorylated *STAT1* and acetylated histone H3 in JAK1/STAT1 leads to IFNG-induced Bcl3 and IL8 expression in OC [201]. LDHB (lactate dehydrogenase B) promotes OC immune escape by enhancing glycolysis-derived lactate, which drives H3K18 lactylation on the PD-L1 promoter, epigenetically upregulating its expression and suppressing T-cell cytotoxicity; inhibition of LDHB restores antitumor immunity and downregulates PD-L1 expression [202]. The histone methyltransferase SETDB1 promotes OC progression by transcriptionally activating SF3B4, a splicing factor that enhances cell proliferation and invasion; SETDB1 also represses antitumor immunity, highlighting its dual role as an epigenetic and immunosuppressive driver in the TME [203].

Overall, histone modifications of inflammatory genes represent critical regulatory mechanisms in OC, with HDACis emerging as promising therapeutic candidates. As research progresses, integrating histone modification targeted therapies with existing chemotherapeutic and immunotherapeutic regimens may pave the way for more effective, durable, and less toxic treatment strategies for OC.

### Non-coding RNAs

Noncoding RNAs (ncRNAs) play a pivotal role in shaping the molecular landscape of OC and are increasingly recognized as central drivers of therapy resistance. Among them, microRNAs (miRNAs), long noncoding RNAs (lncRNAs), and circular RNAs (circRNAs) regulate gene expression at transcriptional, post-transcriptional, and epigenetic levels.

#### MicroRNAs

MicroRNAs (miRNAs) play pivotal roles in regulating inflammatory gene expression in OC, impacting tumor growth, metastasis, and the TME. For instance, miR-23a27a24-2 cluster is crucial for modulating the inflammatory polarization of macrophages. The miR-23a/27a/24 − 2 cluster loss promotes M2 macrophage polarization, which is associated with reduced inflammatory responses and enhanced tumor growth, suggesting that targeting this miRNA cluster could modulate inflammation and serve as a therapeutic approach in OC [204]. Oncosome-derived miRNAs, including miR-222-3p, miR-940, miR-21-3p, miR-125b-5p, miR-181d-5p, and miR-1246, drive macrophage polarization toward the M2 phenotype, which supports tumor progression. TAMs secrete immunosuppressive factors such as TGFB1, VEGFA, IL4, IL5, and IL6, facilitating immune evasion, tumor cell survival, proliferation, invasion, and metastasis [224].

*miR-222-3p* acts as a tumor suppressor in OC by inhibiting CDK19-mediated proliferation, but its function is antagonized by the oncogenic lncRNA *HOTAIR*, which sponges *miR-222-3p* and promotes malignancy through the activation of the *HOTAIR/miR-222-3p*/CDK19 axis [205]. miR-708 exerts antitumor effects by attenuating inflammation, and its induction by low-dose glucocorticoids limits OC progression and metastasis through the suppression of IL1B and IL18 expression and the reduction in the number of immunosuppressive cells in the TME, highlighting its potential as a therapeutic target [206]. miR-217 inhibits M2-like macrophage polarization by decreasing IL6 secretion and attenuating the IL6/JAK/STAT3 signaling pathway, emphasizing its role in regulating inflammation and macrophage function in OC [207]. Dicer is overexpressed in the tumor stroma of OC and transforms normal fibroblasts into a proinflammatory, tumor-promoting state via NF-κB signaling mediated by miR-6780b, thereby facilitating tumor invasion and metastasis [208]. miR-130a, which is overexpressed in HGSOC, promotes inflammation by activating mTOR signaling through the suppression of *TSC1* and is transcriptionally regulated by NF-κB, further contributing to tumor progression and metastasis [209]. Collectively, miRNAs are likely to emerge not only as mechanistic biomarkers for predicting therapeutic response but also as actionable targets for designing more precise and durable treatment interventions.

#### Long noncoding RNAs

Several studies show that long noncoding RNAs (lncRNAs) regulate the expression of inflammatory genes in OC, impacting disease progression and prognosis. For example, lncRNA *MALAT-1* promotes OC progression by activating IL1B and p38/NF-κB/COX2/PGE2 signaling and suppressing IL6 and TNF to promote an immunosuppressive TME while simultaneously enhancing EMT, proliferation, and inhibition of apoptosis [210]. *ZNF667-AS1* (MORT), a tumor suppressor lncRNA that is epigenetically silenced by DNA hypermethylation in OC, promotes proliferation, migration, and invasion. Mechanistically, it exerts these effects through the repression of miRNA-21 as well as the modulation of the TNF signaling pathway [211–213]. The lncRNA *OR3A4* contributes to the inflammatory response in OC by promoting tumor growth and metastasis through the KLF6 pathway. Its knockdown, in combination with cisplatin treatment, enhances anti-inflammatory and antitumor effects [214]. The lncRNA *RUNX1-IT1* and the transcription factor RUNX1 cooperatively drive OC progression via inflammatory and epigenetic mechanisms. *RUNX1-IT1* scaffolds the STAT1-NuRD complex to activate NF-κB through ROS elevation, whereas RUNX1 promotes survival and EMT via the FOXO1-Bcl2 and EGFR-AKT-STAT3 axes; targeting either suppresses metastasis or enhances chemosensitivity [215, 216].

**Table 2:** Gene and protein roles in HGSOC: interconnections with epigenetic and inflammatory pathways

Inflammatory molecule	Epigenetic modifications	Role	References
miR-497	DNA Methylation	Promoter hypermethylation activates mTOR/P70S6K1 and induces cisplatin resistance	[192, 193]
C/EBPβ targets	DNA Methylation + Histone Modification	Altered H3K79 marks promote platinum resistance	[194, 195]
SMAD7	DNA Methylation	Promotes E-cadherin promoter demethylation; restores TGFB signaling and E-cadherin expression	[196].
LINE1	DNA Methylation	Hypomethylation under epigenetic therapy serves as a biomarker for immunotherapy response	[197]
RNF20/H2Bub1	Histone Modification	Loss of H2Bub1 increases IL6 signaling, migration, and tumor initiation	[198]
SIRT1	Histone Modification	Deacetylation enhances immune evasion and reduces PARPi efficacy	[199, 200]
STAT1	Histone Modification	Acetylation via IFNG upregulates IL8 and Bcl3 through the JAK/STAT/NF-κB axis	[201]
LDHB	Histone Modification	H3K18 lactylation at the PD-L1 promoter promotes immune escape via PD-L1 upregulation	[202]
SETDB1	Histone Modification	Histone methyltransferase activity activates SF3B4 and suppresses antitumor immunity	[203]
miR-23a/27a/24 − 2	microRNA	Loss in TAMs enhances M2 polarization and tumor growth	[204]
miR-222-3p	microRNA	Suppressed by *HOTAIR*; regulates CDK19 and affects macrophage polarization	[205]
miR-708	microRNA	Induced by glucocorticoids; inhibits IL1B and IL18, offering anti-inflammatory and anti-metastatic effects	[206]
miR-217	microRNA	Downregulates IL-6/JAK/STAT3; suppresses M2 macrophage phenotype and inflammation	[207]
miR-6780b	microRNA	Activated via NF-κB; induces proinflammatory transformation of fibroblasts	[208]
miR-130a	microRNA	Overexpressed and NF-κB-regulated; suppresses TSC1, activates mTOR, and promotes inflammation	[209]
*MALAT-1*	lncRNA	Activates IL-1β/NF-κB/COX2; suppresses IL6 and TNF, enhancing EMT and immunosuppression	[210]
*ZNF667-AS1* (MORT)	DNA Methylation + lncRNA	Hypermethylated tumor suppressor; represses miR-21 and modulates the TNF signaling pathway	[211–213]
*OR3A4*	lncRNA	Activates KLF6 pathway to promote inflammation, tumor growth, and metastasis	[214]
*RUNX1-IT1*/RUNX1	lncRNA + Transcription Factor	STAT1-NuRD and ROS-mediated activation drives NF-κB signaling, EMT, and chemoresistance	[215, 216]

## Inflammation and chemoresistance

Chemoresistance in HGSOC emerges from the intricate crosstalk between chronic inflammation, tumor-intrinsic plasticity, and the TME. Sustained inflammatory activity perpetuates oxidative stress and cytokine-driven activation of pro-survival cascades, reinforcing transcriptional programs that preserve tumor cell viability and facilitate recovery following cytotoxic insult. Inflammation-responsive genes modulate these adaptive networks, integrating immune and stress-related signaling to maintain drug tolerance, as summarized in (Table 3).

Current management of HGSOC relies on cytoreductive surgery followed by platinum- and taxane-based chemotherapy; however, therapeutic efficacy is constrained by the frequent development of resistance. Intrinsic resistance is attributed to pre-existing molecular configurations within chemo-naïve tumor cells, whereas acquired resistance results from drug-induced transcriptional and epigenetic remodeling that enhances cellular survival [235]. Tumor cells evade chemotherapy through elevated efflux capacity, reduced drug uptake, metabolic inactivation, augmented DNA repair, and suppression of apoptotic signaling [236].

Within the TME, stromal-tumor interactions critically modulate both intrinsic and adaptive resistance mechanisms. Environment-mediated drug resistance (EMDR) and soluble factor-mediated drug resistance (SFM-DR) exemplify microenvironment-dependent adaptations. In SFM-DR, fibroblast-derived IL6 activates STAT3 signaling, leading to transcriptional upregulation of anti-apoptotic regulators such as Bcl-xL, Bcl-2, and XIAP, thereby diminishing platinum sensitivity [21, 237]. Cell adhesion-mediated drug resistance (CAM-DR) similarly arises through integrin-dependent interactions between tumor cells and extracellular matrix (ECM) proteins, including laminin, collagen, and fibronectin, which transiently induce a drug-tolerant state independent of intracellular drug accumulation [238].

Chronic inflammatory conditions further potentiate resistance by generating a cytokine- and ROS-enriched milieu that supports metabolic adaptation and tumor persistence [239, 240]. ROS activate PGC-1α-dependent mitochondrial biogenesis, promoting expression of ALDH and MDR1, which collectively enhance detoxification and drug efflux capacity [241]. Likewise, LPA signaling facilitates chemoresistance by inducing EMT and inhibiting autophagic flux, thereby mitigating platinum-induced apoptosis [242].

**Table 3:** Inflammation-associated genes and their roles in the chemoresistance mechanisms of HGSOC

Gene	Regulation (up/down)	Role	References
CCL5	Upregulated	Functions as a chemoattractant and promotes cisplatin resistance through activation of STAT3 and the PI3K/Akt pathway.	[225]
CD70	Upregulated	A TNF-family ligand, found in higher levels in platinum-resistant cells, associated with drug resistance and reduced survival outcomes.	[226]
IL6	Upregulated	Enhance resistance to platinum-based therapy by triggering STAT3 signaling and contributing to autocrine feedback loops.	[85, 227]
CXCL12	Downregulated	Involved in EMT and STAT3 activation; its reduced expression is linked to platinum resistance.	[165, 228]
IL8	Upregulated	Secreted by OC cells, IL8 contributes to chemoresistance by lowering caspase-3 activation and promoting anti-apoptotic signaling through pathways like PI3K/Akt and MEK/ERK.	[229]
CXCR2	Upregulated	Stimulates tumor growth, angiogenesis, metastasis, and therapy resistance via its role as a receptor for pro-inflammatory chemokines.	[230]
TGFB	Upregulated	Plays a multifaceted role in resistance by promoting EMT, cancer stemness, and hypoxia adaptation via HIF-2α, reinforcing a resistant TME.	[40, 231]
CCL2	Downregulated	Reduced expression in resistant HGSOC tumors is associated with decreased immune cell recruitment and enhanced tumor aggressiveness, suggesting a complex role in disease progression.	[232]
TNF	Upregulated	Activates NF-κB signaling, facilitating cancer cell survival, EMT induction, and resistance to chemotherapy.	[233]
IFNG	Upregulated	IFNG can inhibit tumor growth via SOCS1/JAK/STAT signaling and promote apoptosis, its overexpression may also trigger immune tolerance and reduce chemosensitivity.	[234]

## Inflammatory genes and epigenetic modifications contributing to chemoresistance

Chronic inflammation plays a pivotal role in driving epigenetic modifications during the progression of HGSOC. Persistent inflammatory signaling mediated by cytokine and inflammatory mediators, induces alterations in DNA methylation, histone modification, and non-coding RNA expression, leading to transcriptional reprogramming that underlies intrinsic chemoresistance. Therapeutic interventions such as platinum-based chemotherapy can further exacerbate these changes, reactivate inflammatory pathways and establish a self-perpetuating cycle of inflammation and epigenetic remodeling that drives tumor recurrence and treatment failure.

Beyond the canonical cytokine-receptor signaling, epigenetic reprogramming of inflammatory mediators provides a crucial link between chronic inflammation to transcriptional plasticity in HGSOC. Continuous exposure to proinflammatory cytokines such as IL6, TGFB, TNF, and IL1B sustains chromatin alternations that stabilizes oncogenic transcriptional states even after cessation of the inflammatory stimulus [243]. Furthermore, inflammation-responsive miRNAs and lncRNAs modulate these inflammatory pathways, reinforcing transcriptional adaptability and sustained chemoresistance [244, 245].

At the genome-wide level, global DNA hypermethylation, coupled with site-specific CpG methylation of *ITGB6* and *NCALD*, has been associated with poor prognosis and chemoresistance in HGSOC, reflecting decreased mRNA and protein expression of adhesion and signaling molecules crucial for chemosensitivity [246]. Furthermore, loss of 5-hydroxymethylcytosine (5hmC), secondary to *TET2* downregulation, has emerged as a hallmark of platinum resistance and tumor aggressiveness. Restoration of 5hmC through 5-azacytidine treatment resensitizes resistant cells, highlighting the therapeutic reversibility of these epigenetic lesions [247, 248].

Epigenetic dysregulation also extends to histone demethylases, particularly JMJD2A (KDM4A), which is markedly upregulated in ovarian cancer tissues. JMJD2A demethylates H3K9me3/2 and H3K36me3/2, resulting in transcriptional activation of *IL6* and *IL8*, thereby driving oncogenic inflammation, cell proliferation, and resistance to platinum-based chemotherapy [249]. This establishes a feed-forward inflammatory circuit, reinforcing STAT3 and NF-κB activation. Moreover, oxidative stress induced by chronic inflammation further disrupts histone modification patterns and modulates microRNAs targeting tumor suppressor genes (*BRCA1/2*, *TP53*, *PTEN*, *PIK3CA*), thereby fostering EMT, enhanced DNA repair, and acquisition of stem-like properties [250]. These events not only sustain inflammation but also promote the progression of endometriosis-associated lesions into chemo-resistant HGSOC phenotypes.

Within the TME, exosomal miR-21 derived from cancer-associated fibroblasts (CAFs) epigenetically represses the proapoptotic gene *APAF1* in HGSOC cells, thereby enhancing resistance to platinum compounds and amplifying IL-6/STAT3 signaling loops that reinforce paracrine inflammatory communication between stromal and tumor compartments [251]. Similarly, epigenetic silencing of *YWHAB* (14-3-3β) disrupts its inhibitory control over YAP, a downstream effector of the Hippo pathway that regulates proliferation and apoptosis. Loss of YWHAB-mediated repression leads to unchecked YAP nuclear translocation and activation of transcriptional programs that drive cancer cell stemness, metabolic reprogramming, and inflammatory gene expression, collectively promoting chemoresistance and tumor recurrence [252–254].

These findings highlight the critical roles of inflammatory genes and epigenetic changes in the development of chemoresistance in HGSOC, opening new avenues for more precise and effective treatment approaches.

## Treatment strategies focused on inflammation and epigenetics

Translating the mechanistic understanding of inflammation-epigenetic interplay in HGSOC into effective therapies has become a central focus in recent years. These intersecting pathways not only underpin tumor aggressiveness and immune evasion but also shape chemoresistance, providing a strong rationale for therapeutic co-targeting.

Epigenetic agents such as DNMT and HDAC inhibitors have demonstrated the ability to reverse transcriptional silencing of tumor-suppressor and immune-modulation in preclinical models [255]. Guadecitabine, a DNMT inhibitor, combined with carboplatin, has shown partial reactivation of DNA repair and apoptotic pathways in platinum-resistant ovarian cancer, though gains in progression-free survival remain limited likely reflecting transient epigenetic reprogramming and intertumoral heterogeneity [256]. Similarly, class I-selective HDACis targeting HDAC3 influence inflammatory cytokine signaling and apoptotic responses, but their application is constrained by dose-limiting toxicity, incomplete isoform selectivity leading to broad histone-independent effects [257]. Among combinatorial strategies, dual targeting of epigenetic regulators and DNA repair pathways has shown preclinical promise. PARPi combined with EZH2 or G9A inhibition activate interferon-responsive programs, upregulating immune-stimulatory chemokines (CXCL9, CXCL10) and transcriptional regulators (STAT1, IRF7), with associated modulation of the tumor immune microenvironment [258, 259]; however, clinical translation remains limited by context dependency, toxicity, and inadequate biomarker stratification.

Targeting the immuno-epigenetic axis represents another promising approach. In *TP53*-mutant tumors, GATA3-enriched tumor-associated macrophages promote M2 polarization and EMT via IL10, CCL18, and TGFB signaling, thereby driving metastasis and chemoresistance. Pharmacologic inhibition of GATA3 or disruption of TLR4-MyD88-NF-κB signaling (e.g., TAK-242) restores chemosensitivity by attenuating this inflammatory crosstalk [260, 261]. Similarly, epigenetic reactivation of RGS10 through DNMT and HDAC inhibition suppresses TNFA and COX2 expression, enhancing apoptotic susceptibility and limiting prostaglandin-mediated survival signaling [262, 263]; however, the therapeutic potential of this approach may be constrained by the non-specific effects of epigenetic drugs, tumor heterogeneity, and the reversible nature of epigenetic modifications, warranting further in vivo validation to confirm its clinical relevance and safety.

Anti-inflammatory agents such as aspirin further exemplify this therapeutic convergence. By inhibiting platelet-derived thromboxane A₂, aspirin enhances T-cell activation and limits metastatic potential [253]. Phosphatidylcholine-conjugated aspirin (aspirin-PC) demonstrates superior antitumor activity and reduced gastrointestinal toxicity, particularly when combined with VEGF inhibitors [254]. Aspirin also suppresses EGF-driven Akt/Erk signaling in COX1 positive ovarian cancer and enhances p53 acetylation, upregulating tumor-suppressor genes including *CDKN1A*,* BAX*,* FOXF1*,* PUMA*, and *RRAD*, thereby improving cisplatin responsiveness [255, 256]. Despite decades of preclinical and epidemiologic promise, its translation to standard adjuvant therapy remains limited by toxicity constraints (which may be mitigated through conjugated-drug formulations), variability in COX1 expression, and the absence of large-scale, biomarker-stratified clinical validation.

## Combination therapies

**Fig. 5:**
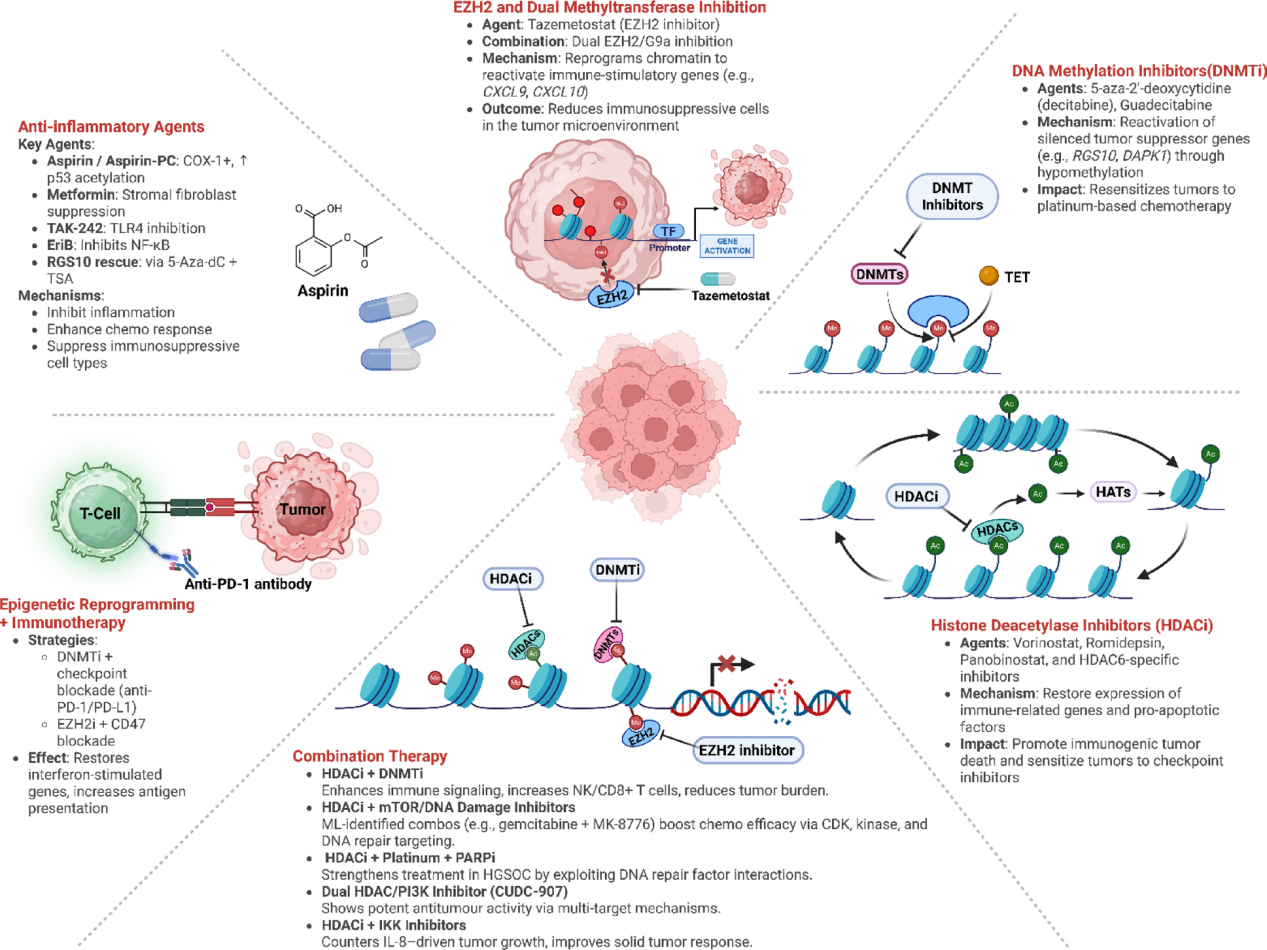
Epigenetic reprogramming and combination therapies: mechanisms and agents in ovarian cancer. Overview of rational combination therapy strategies in OC that integrate epigenetic modulators, anti-inflammatory agents, and DNA damage response inhibitors to overcome resistance and enhance therapeutic efficacy. Created in BioRender. https://BioRender.com/r24e57n

Recent investigations have advanced the paradigm of rational combination therapy by integrating epigenetic modulators, chemotherapeutics, and immune-targeted or anti-inflammatory agents to overcome the adaptive resistance networks in HGSOC (Fig. 5). Rather than acting as independent agents, these therapies rewire the tumor immune microenvironment, modulate cytokine signaling, and enhance DNA repair vulnerability creating conditions conducive to durable therapeutic response.

For instance, combining the DNMT inhibitor 5-Aza-2′-deoxycytidine (5-Aza-dC) with *ADAR1* knockdown has been shown to augment type I IFN signaling, increase secretion of proinflammatory chemokines such as CCL5, and enhance CD8⁺ T-cell infiltration [264]. This approach not only reactivates silenced endogenous retroviral elements but also counteracts immune evasion mechanisms that are typically reinforced by the inflammatory TME. These findings underscore that epigenetic priming can convert “immune-cold” ovarian tumors into immunologically active phenotypes, thereby potentiating immunotherapy efficacy.

Dual inhibition of DNMT and histone deacetylase 6 (HDAC6) further reinforces this concept. Such co-targeting amplifies type I IFN signaling and antigen presentation via MHC class I, while simultaneously enhancing the infiltration of NK and CD8⁺ T cells, effectively reprogramming the immunosuppressive TME [265]. These immunostimulatory effects have translated into prolonged survival in preclinical models of chemoresistant OC, highlighting the potential of epigenetic-immune co-modulation as a mechanism to restore antitumor responsiveness. However, the translational challenge remains in balancing immune activation with systemic toxicity, as heightened cytokine induction can provoke adverse inflammatory responses in clinical contexts.

A similar mechanistic rationale underpins the guadecitabine-pembrolizumab combination in platinum-resistant OC, where demethylation of LINE-1 elements by guadecitabine led to the re-expression of immune-activating genes, including *IFNG*, *CXCL9*, and *IL21* ​ [266]. Although this epigenetic reactivation enhanced interferon-driven immune signaling, compensatory upregulation of inhibitory checkpoints such as LAG3, IDO1, and A2AR emerged highlighting an intrinsic feedback resistance mechanism. This underscores the necessity for triplet combinations that include immune checkpoint blockade alongside epigenetic priming to mitigate immunosuppressive counter regulation and sustain antitumor immunity.

The synergistic interaction of HDAC6 inhibition with DNA damage repair agents, including platinum compounds and PARPi, has also demonstrated therapeutic potential in HGSOC. Mechanistically, HDAC6 inhibition disrupts microtubule dynamics and DNA damage response fidelity, thereby enhancing the cytotoxic efficacy of platinum drugs [267]. However, given the narrow therapeutic window of HDAC6 inhibitors and their potential to influence non-histone substrates involved in DNA repair, the optimization of dosing and scheduling remains a key translational barrier.

Among dual-pathway inhibitors, CUDC-907, which concurrently targets PI3K-AKT and HDAC-c-Myc signaling, exemplifies an integrated approach to suppressing both metabolic and epigenetic dependencies in OC. This agent induces apoptosis, cell-cycle arrest, and H3K9 acetylation, while simultaneously repressing oncogenic c-Myc and Bcl-2 signaling [268]. Such polypharmacologic interventions are particularly appealing in inflammation-driven tumors like HGSOC, where crosstalk between survival and epigenetic circuits sustains therapeutic resistance. Nevertheless, the clinical challenge lies in disentangling the dominant pathway drivers in individual tumors to ensure precision in combination design.

Despite the therapeutic promise of HDACis, their use in solid tumors is complicated by paradoxical activation of proinflammatory mediators such as IL8 via IKK (IκB kinase), which inadvertently fosters tumor growth. The combination of HDAC and IKK inhibitors has therefore emerged as a more rational approach, potentially reversing this protumorigenic feedback loop while maintaining the cytostatic effects of HDAC inhibition [269]. This strategy underscores the critical importance of contextual drug pairing, where the downstream immune and cytokine consequences of epigenetic therapy are anticipated and pharmacologically counterbalanced.

Advances in machine learning (ML) have further accelerated the identification of synergistic drug pairs with complex mechanistic interplay. Computational models integrating transcriptomic and phosphoproteomic datasets have predicted high-efficacy combinations such as HDACis with mTOR and DNA damage response inhibitors, or gemcitabine with MK-8776, and dinaciclib or AZD1775 with MK-8776, which collectively enhance chemotherapy sensitivity by targeting CDK regulation, kinase phosphorylation, and DNA repair pathways [270]. ML-driven frameworks provide a systematic approach to rational combination design, replacing traditional trial-and-error screening with mechanism-based predictive analytics.

Computational and ML-based frameworks are reshaping combination therapy development in HGSOC by predicting homologous recombination deficiency (HRD) and refining regimens that target inflammation-driven chemoresistance and epigenetic reprogramming. *DeepHRD*, a ResNet18 CNN ensemble with multiple-instance learning on H&E slides, identifies HRD signatures across *BRCA1/2*,* PALB2*, and *RAD51* from TCGA, while detecting 1.8–3.1 fold more HRD-positive cases than genomic assays [271]. Immune-related ML stratification of TCGA RNA-seq data using random forest modeling identified distinct TME phenotypes with divergent immune signaling and survival outcomes, highlighting the potential of immune-modulatory strategies in HGSOC; however, its dependence on retrospective data and absence of experimental validation underscore the need for prospective, multi-omic studies to confirm clinical relevance [272]. Integration of multimodal ML approaches has enhanced the precision of treatment selection in HGSOC by leveraging genomic, radiomic, and histopathologic data to refine risk stratification and predict patient-specific responses to platinum-based and combination therapies, outperforming conventional predictive methods [273]. Evidence demonstrates that ML algorithms, particularly support vector machines (SVMs), effectively identify platinum-resistant phenotypes, supporting their use in designing combination regimens that integrate platinum agents with targeted or immune-based therapies [274]. Additionally, proteomic ML analyses have uncovered key biomarkers such as TOP1, PDIA4, and OGN, which are implicated in chemoresistance pathways and represent potential targets for dual cytotoxic-molecular treatment strategies [275]. Broader oncologic evaluations of ML-based frameworks further indicate their potential in enabling adaptive and individualized combination regimens across malignancies [276]. Despite these advancements, current evidence is primarily derived from retrospective analyses, underscoring the need for prospective, multi-center validation to establish clinical reliability and translate ML-driven treatment paradigms into real-world practice.

Advances in single-cell and multi-omic technologies have unraveled the intertwined inflammatory, metabolic, and epigenetic programs that underpin therapeutic resistance in HGSOC. Using computational tools like- *InferCNV*, *Monocle*, *CytoTRACE*, *Slingshot*, and *CellChat*, epithelial heterogeneity and MK/MDK-NCL mediated fibroblast crosstalk were mapped, defining inflammation-driven glycolytic subtypes [277]. Multi-layer integration of SCNA, methylation, chromatin, and transcriptomic data revealed key regulatory networks (*FOXK1*,* NFE2L2*,* CCN1*,* HSP90AA1*), while transcriptome-methylome coupling through consensus clustering and machine-learning feature selection identified distinct molecular states (HRD/OXPHOS and BMI-1) associated with immune suppression and recurrence [278, 279]. Together, these frameworks demonstrate that resolving inflammatory-epigenetic crosstalk demands robust computational harmonization, providing a blueprint for precision therapeutic targeting in advanced HGSOC.

Liquid biopsy driven approaches are redefining therapeutic monitoring and management in HGSOC by enabling longitudinal, non-invasive molecular profiling of tumor evolution. Circulating analytes such as cfDNA, ctDNA, CTCs, and exosomes enable real-time monitoring of treatment response and resistance, offering greater temporal sensitivity than conventional markers [280]. Quantitative assessment of cfDNA and ctDNA using droplet digital PCR (ddPCR) has demonstrated clinical relevance in evaluating cytoreductive efficacy and predicting recurrence, with molecular perturbations preceding radiologic progression [281]. In parallel, methylation-based cfDNA assays such as *OvaPrint*, developed through reduced-representation bisulfite sequencing and hybrid-capture enrichment, achieved > 90% diagnostic accuracy, supporting early molecular stratification and treatment planning [282]. Collectively, these modalities form an integrated framework for adaptive, biomarker-guided therapy in HGSOC, though translation into clinical practice remains limited by assay standardization, early-stage detection sensitivity, and the need for prospective validation linking molecular trajectories to therapeutic outcomes.

## Limitations and future perspectives

Despite substantial advances in decoding the inflammatory-epigenetic-chemoresistance axis in HGSOC, key translational and methodological constraints continue to hinder clinical translation. Current bulk-tissue analyses inadequately capture HGSOC’s extensive spatial and temporal heterogeneity, obscuring how IL6/STAT3 and NF-κB signaling remodels chromatin to establish a stable “epigenetic memory” a heritable transcriptional state that maintains the inflammation-induced survival and drug-resistant phenotypes even after the initial stimuli subside. Longitudinal single-cell multi-omic and spatial epigenomic mapping are therefore essential to unravel these dynamic transitions. Therapeutically, DNMT and HDAC inhibitors can reverse silenced gene networks but are constrained by pharmacokinetic liabilities, toxicity, and compensatory PI3K/AKT activation which restores pro-survival signaling. Co-targeting the IL6/STAT3 axis with PARPi or epigenetic agents offers a mechanistically robust strategy to dismantle inflammation-driven adaptive resistance. Integration of methylomic and cytokine biomarkers with machine learning based patient stratification could refine therapeutic selection, optimize dosing, and enable dynamic response monitoring in HGSOC.

The IL6/STAT3 axis emerges as a mechanistically compelling target for HGSOC combination therapy, centrally driving chemoresistance, cancer stemness, and inflammation-induced epigenetic reprogramming. STAT3 not only orchestrates survival and immune evasion programs but directly recruits DNMTs, EZH2, and HDACs to epigenetic machinery, thereby stabilizing drug-resistant phenotypes. Despite robust preclinical rationale, clinical translation of STAT3-epigenetic combinations remains limited by suboptimal pharmacokinetics and toxicity of early-generation STAT3 inhibitors, context-dependent pleiotropic STAT3 signaling across tumor/immune compartments [283], overlapping epigenetic drug safety concerns [284]. and absent validated biomarkers for STAT3-addicted, epigenetically primed tumors. Biomarker-enriched trial designs and next-generation selective STAT3 inhibitors offer the path to clinically viable chemoresistance reversal.

AI-driven drug discovery remains limited by insufficient modeling of tumor–stroma crosstalk and phenotypic plasticity; incorporating spatial transcriptomics, proteomic flux analysis, and organoid-based validation will enhance predictive relevance. Similarly, refinement of anti-inflammatory adjuvants such as aspirin and TLR4 inhibitor TAK-242 requires AI-assisted pharmacodynamic modeling to balance efficacy and safety.

Ex vivo functional assays including organoids, explants, and primary tumor cultures are emerging as pivotal tools in precision oncology, enabling direct assessment of drug sensitivity in patient-derived tumor models [285, 286]. Unlike conventional genomics-based profiling these platforms preserve tumor heterogeneity, microenvironmental context, and phenotypic plasticity, providing a more accurate therapeutic efficacy [287]. Delivering high-throughput results within days, they have achieved clinical response rates of up to 88% from 59% in hematologic and solid malignancies and have guided off-label therapeutic use with sustained disease control [285]. When integrated with apoptotic priming assays like BH3 profiling, these systems can reveal targetable anti-apoptotic vulnerabilities, improving precision and reducing empirical treatment cycles [288]. In the context of ovarian cancer, where intratumoral heterogeneity and treatment resistance remain major challenges, such ex vivo functional diagnostics hold significant promise for bridging molecular insights with clinically actionable precision therapy.

Future research should converge toward a next-generation systems-oncology framework that integrates real-time biomarker analytics, AI-enabled drug discovery, and adaptive clinical trial design. By coupling longitudinal molecular profiling with dynamic therapeutic feedback, such an approach would enable precise modulation of the inflammatory-epigenetic circuitry that drives therapeutic escape. This paradigm aims not merely to extend transient responses but to reprogram the chemoresistant tumor microenvironment, thereby achieving durable, mechanism based clinical remission in HGSOC.

## Conclusion

HGSOC represents a highly heterogeneous malignancy in which chronic inflammation serves as a central molecular driver rather than merely as a secondary consequence. Sustained activation of key inflammatory signaling pathways such as IL6/STAT3 and NF-κB, promotes tumor proliferation, immune evasion, and therapeutic resistance by establishing stable, heritable epigenetic modifications. Targeting this intertwined inflammatory-epigenetic axis necessitates rationally designed combinatorial strategies, particularly those coupling HDAC and DNMT inhibitors with immunotherapeutic approaches, to disrupt the self-reinforcing oncogenic feedback loop. Given the intricate and evolving molecular nature of HGSOC, optimizing such interventions will rely on precision patient stratification through multi-omics, liquid biopsy based on methylomic/cytokine profiling, and machine learning driven analytics to enable adaptive, personalized treatment paradigms that reprogram immune-cold tumors into immune-responsive phenotypes capable of achieving sustained clinical remission.

## Data Availability

No datasets were generated or analysed during the current study.

## Abbreviations

5hmC: 5-Hydroxymethylcytosine
CRP: C-reactive protein
DNMT: DNA methyltransferase
EOC: Epithelial ovarian cancer
EMT: Epithelial-mesenchymal transition
HGSOC: High-grade serous ovarian cancer
HDAC: Histone deacetylase
HDACis: Histone deacetylase inhibitors
IFNs: Interferons
IL1: Interleukin 1
IL6: Interleukin 6
IL8: Interleukin 8
FGF: Fibroblast growth factor
lncRNAs: Long noncoding RNAs
miRNAs: MicroRNAs
MDSCs: Myeloid-derived suppressor cells
NF-κB: Nuclear factor kappa-light chain enhancer of activated B cells
OC: Ovarian cancer
PARPi: PARP inhibitors
LPA: Phospholipid lysophosphatidic acid
ROS: Reactive oxygen species
Bregs: Regulatory B cells
TGFB: Transforming growth factor-beta
TME: Tumor microenvironment
TNF: Tumor necrosis factor
TAMs: Tumor-associated macrophages
TANs: Tumor-associated neutrophils
Tregs: Regulatory T cells
VEGF: Vascular endothelial growth factor
